# Dietary Effects of a Short-Term Administration of Microalgae Blend on Growth Performance, Tissue Fatty Acids, and Predominant Intestinal Microbiota in *Sparus aurata*

**DOI:** 10.3390/microorganisms11020463

**Published:** 2023-02-12

**Authors:** Jorge García-Márquez, Rosa María Rico, Francisco Gabriel Acién, Juan Miguel Mancera, Félix L. Figueroa, Antonio Jesús Vizcaíno, Francisco Javier Alarcón, Miguel Ángel Moriñigo, Roberto Teófilo Abdala-Díaz

**Affiliations:** 1 Departamento de Microbiología, Facultad de Ciencias, Instituto Andaluz de Biotecnología y Desarrollo Azul (IBYDA), Universidad de Málaga, Ceimar-Universidad de Málaga, 29071 Málaga, Spain; 2 Departamento de Ecología y Geología, Facultad de Ciencias, Instituto Andaluz de Biotecnología y Desarrollo Azul (IBYDA), Universidad de Málaga, Ceimar-Universidad de Málaga, 29071 Málaga, Spain; 3 Departamento de Ingeniería Química, Universidad de Almería, Ceimar-Universidad de Almería, 04120 Almería, Spain; 4 Departamento de Biología, Facultad de Ciencias del Mar y Ambientales, Instituto Universitario de Investigación Marina (INMAR), Universidad de Cádiz, Ceimar-Universidad de Cádiz, 11510 Cádiz, Spain; 5 Departamento de Biología y Geología, Universidad de Almería, Ceimar-Universidad de Almería, 04120 Almería, Spain

**Keywords:** fatty acid composition, microbiota, *Tisochrysis lutea*, *Nannochloropsis gaditana*, *Scenedesmus almeriensis*, *Sparus aurata*

## Abstract

Given the potential of microalgae as new aquafeed ingredients, this study focuses on using a blend of microalgae, *Tisochrysis lutea*, *Nannochloropsis gaditana*, and *Scenedesmus almeriensis*, as a dietary ingredient for feeding *Sparus aurata* juveniles. The growth performance, carcass composition, tissue fatty acid profile, and intestinal microbiota were evaluated after a 30 day-feeding period. A microalgae-free diet was used as control, and three experimental diets were formulated containing 5%, 15%, and 25% of the microalgae blend (MB-5%, MB-15%, and MB-25%, respectively). After 7, 15, and 30 days of feeding experimental diets, biological samples were taken. Growth performance and nutrient utilization were not significantly modified at the end of the experiment. Microalgae inclusion tended to decrease body lipids and affected the fatty acid profile, especially MB-25 diet increased DHA levels. Diet MB-25 promoted appropriate microbial diversity, favoring the presence of probiotic bacteria, such as *Lactobacillus*, and significantly influencing the fatty acid composition and lipid metabolism in fish. In conclusion, using a short pulse of dietary administration of 25% microalgal blend in *S. aurata* modulates the intestinal microbiota and lipid composition while maintaining growth performance.

## 1. Introduction

Aquaculture plays a relevant role in providing high-quality protein and lipids for human consumption. However, the culturing of aquatic animals involves a high amount of protein in their diets. Dietary protein is obtained mainly from captured wild fish or crop cultures, making aquaculture expansion based on fishmeal and land use unsustainable [1,2]. Thus, expanding the aquafeed industry requires finding sustainable ingredients [3]. The major challenges for finding suitable alternative protein sources in aquafeeds are the fluctuating protein content in those feedstuffs and the practicality of expanding their production, which are dependent on current processing technologies and scalability while promoting additional nutritional health advantages [4].

Microalgae have the potential to replace fishmeal and fish oil in aquafeeds because they provide high-quality protein and a noticeable amount of essential amino acids [5,6,7]. Furthermore, although there are strain- and species-specific differences in fatty acid content, some microalgae may be considered a viable source of polyunsaturated fatty acids (PUFA), particularly eicosapentaenoic and docosahexaenoic acids (EPA and DHA, respectively) [8].

Many studies have shown that microalgae can be used as a dietary ingredient in different aquaculture species, generally without a detrimental impact on growth and general health [9,10,11]. Despite this, the most significant limitation to the widespread use of microalgae is that any acceptable alternative feed ingredient has to provide adequate nutritional content at a competitive cost. However, this last still needs to be achieved by employing algae as a major component in aquafeeds, particularly in the case of microalgae [12].

The present work focuses on using a blend of microalgae, *Tisochrysis lutea*, *Nannochloropsis gaditana*, and *Scenedesmus almeriensis*, as a dietary ingredient for feeding *Sparus aurata* juveniles. The potential of these microalgae species for use individually as dietary ingredients has been previously assessed in previous studies performed by our research group [10,13,14,15]. The microalga *T. lutea* is considered a promising feed ingredient owing to its content in fatty acids, especially docosahexaenoic acid (DHA), up to 203 mg g^−1^ (dry biomass) total fatty acids. DHA plays key roles in the health and development of aquatic organisms, and this strain is widely used in aquaculture feeds [16]. *N. gaditana* is of particular interest to the aquafeed industry for the production of high-value oil containing omega-3 fatty acids, specifically eicosapentaenoic acid (EPA), ranging from 70 to 380 mg g^−1^ dry biomass [17]. *S. almeriensis* has huge potential to achieve commercial success due to its high contents of protein and lutein; this strain has been suggested as a potential functional feed ingredient showing potential for use as a feed ingredient promoting the gut functionality of fish [15]. The blend of those microalgae contains high amounts of protein and an interesting fatty acid profile that includes significant levels of α-linolenic acid, EPA, and DHA [18,19]. 

To the best of our knowledge, no studies are available evaluating the effect of dietary administration of that microalgal blend during a short feeding period as a functional feed ingredient in seabream juveniles. The objective of the present study was to evaluate the effect of different dietary levels of a microalgae blend composed of 33.3% *Tisochrysis lutea*, 33.3% *Nannochloropsis gaditana*, and 33.3% *Scenedesmus almeriensis* on growth performance, nutrient utilization, body composition, fatty acid profile, and intestinal microbiota in *S. aurata* juveniles.

## 2. Materials and Methods

### 2.1. Ethical Statement

Fish were kept and handled following the guidelines for experimental procedures in animal research from the Ethics and Animal Welfare Committee of the University of Cadiz, according to the Spanish (RD53/2013) and European Union (2010/63/UE) legislation.

### 2.2. Microalgae Biomass

The microalgae were cultured in photobioreactors at the pilot plant (EU-H2020 SABANA facilities funded by grant #727874) of the Universidad de Almería (Spain). The biomass was produced in tubular photobioreactors (PBR) (3000-L water capacity, 0.09 m tube diameter) with automatic temperature control. The pH, temperature, and dissolved oxygen values were continuously monitored at the end of the loop using specific probes (Crison Instruments, Alella, Spain). pH was controlled automatically by injection of CO_2_ at the beginning of the loop. The temperature was kept within the range required for optimal growth of each microalgae strain (*T. lutea* 23–25 °C, *N. gaditana* 25–27 °C, and *S. almeriensis* 28–30 °C) by passing cold/hot water through a heat exchanger located inside the bubble column of the reactor. The culture medium used was Mann and Myer [20], which was prepared by dissolving fertilizers in irrigation water and then sterilizing by filtration/ozone. Microalga biomass was harvested by centrifugation (OTC3, CEA Westphalia, Oelde, Germany), frozen at −20 °C, and lyophilized. Dry biomass was subsequently milled and sieved through a 50 µm pore to obtain a homogenized powder that was stored in the dark at −20 °C until further preparation of the experimental diets. 

### 2.3. Experimental Feeds and Feeding Trial

The experimental diets were manufactured at the Servicio de Dietas Experimentales of the Universidad de Almería (http://www.ual.es/stecnicos_spe, accessed on 20 December 2022). For this, standard aquafeed procedures, i.e., mixing ingredients, the inclusion of feed additives, gentle extrusion with temperature control, and extrusion within the size range of experimental feed, consisting of four isoproteic (45%) and isolipidic (10%) inert diets (2.0 mm), were used. The algae-supplemented diet was formulated containing a 5%, 15%, and 25% of microalgae blend composed by 33.3% *T. lutea* (for providing protein and DHA), 33.3% *N. gaditana* (for providing protein and EPA), and 33.3% *S. almeriensis* (for providing protein and linolenic acid) (MB-5, MB-15, and MB-25, respectively). A control diet was formulated without microalgae. The ingredient composition, nutrient composition, and the fatty acid profile of the experimental diets are presented in Table 1 and Table 2, respectively. 

Juvenile specimens of *S. aurata* were obtained from CUPIMAR (San Fernando, Cádiz, Spain). The feeding trial was conducted at the Servicios Centrales de Investigación en Cultivos Marinos (SCI-CM, CASEM, University of Cádiz, Puerto Real, Cádiz, Spain; Spanish Operational Code REGA ES11028000312). After arrival at the experimental unit, fish were conditioned in a 1000-L tank (4 tanks, 50–55 fish per tank) and acclimated in an open marine water circuit for two weeks. During the adaptation period, fish were fed a commercial diet (48% protein, 25% lipids, 11.5% ash, 20.2 kJ g^−1^; Skretting, L2 Active 1P, Skretting, Spain).

Fish (n = 144, 16.1 ± 0.3 g body mass, mean ± SD) were randomly distributed in a flow-through setup of twelve 100 L tanks to establish four experimental groups in triplicate. Fish were maintained under natural photoperiod (July–August, 14:10 h, light:dark, LD; 36°31′45″ N, 6°11′31″ W), temperature (18–19 °C) and salinity (38–39 ppt.). Fish were hand fed twice daily (9:00 and 17:00) at a rate of 2.5% of their body weight over 30 days with the experimental feeds. The uneaten pellets were collected after 1 h and then dried and weighed. Supplemental aeration was provided to maintain dissolved oxygen at 6.8 ± 0.4 mg L^−1^. Ammonia (<0.1 mg L^−1^), nitrite (<0.2 mg L^−1^), and nitrate (<50 mg L^−1^) were determined once weekly at 9 a.m.

### 2.4. Fish Sampling

Animals were anesthetized with 2-phenoxyethanol (0.5 mL L^−1^ SW, Sigma-Aldrich #77699, St. Louis, MI, USA) at the beginning of the assay (day 0) and on days 7, 15, and 30. Fish were individually weighed, and the daily feed rations were adjusted accordingly to maintain the initial 2.5% rate of their body mass during the experiment. At every sampling point (days 0, 7, 15, and 30), animals were taken from their respective tanks (two fish per tank × three tanks per diet) after 24 h of feed deprivation, and then euthanized by an overdose of 2-phenoxyethanol (1 mL L^−1^ SW) followed by spine severing according to the requirements of the EU Directive 2010/63/EU for animal experiments. At day 30, the body weight of the remaining animals was recorded, and growing parameters were calculated as indicated in Section 2.5 (see below in the text). The abdomen was opened, and the digestive tract and liver were removed and weighed to determine viscerosomatic and hepatosomatic indices (VI and HSI, respectively). The carcasses were freeze-dried and stored at −80 °C for further proximate composition analysis. Whole intestines were aseptically removed and kept at −80 °C for microbiota analysis. Additionally, at the end of the experiment (day 30), the muscle and liver were aseptically removed and kept at −80 °C for lipid and fatty acid analysis.

### 2.5. Growth Performance, Nutrient Utilization, and Somatic Indices

To evaluate the growth performance and nutrient utilization, we used the following equations (Equations (1)–(8)):

Weight Gain (WG, %) = ((final fish weight − initial fish weight) × 100)(1)

Specific Growth Rate (SGR, % day^−1^) = (100 × [(ln final fish weight) − (ln initial fish weight)]/experimental days) (2)

Feed Conversion Ratio (FCR) = dry feed intake (g)/weight gain (g)(3)

Protein Efficiency Ratio (PER) = weight gain/intake of particular protein(4)

Carcass Yield (CY, %) = ((carcass weight/body weight) × 100)(5)

Condition Factor (K, %) = ((fish weight/fish length^3^) × 100) (6)

Viscerosomatic Index (VI, %) = ((visceral weight (g)/whole body weight (g)) × 100)(7)

Hepatosomatic Index (HSI, %) = ((liver weight/body weight) × 100)(8)

### 2.6. Chemical Composition of Algae and Fish

Total carbon and nitrogen were determined from the dry biomass of microalgae and the liver and muscle of specimens using a CNHS LECO-932 elemental analyzer (Michigan, USA) in the Research Support Central Services (SCAI, University of Malaga, Spain). Total proteins were calculated from the elemental N determination using the N-protein conversion factor [21]. Lipids were extracted according to the method described by Folch et al. [22]. The sample (150–200 mg) was homogenized in chloroform:methanol (2:1) for 5 min. The lipid fraction was separated by centrifugation, and total lipid content was calculated by gravimetry once the solvent (chloroform) was evaporated entirely from the lipid fraction. The dry matter content was determined after drying the samples in an oven at 110 °C until constant weight. The ash content was determined by combustion of the sample to constant weight in a muffle oven at 600 °C for 12 h.

### 2.7. Fatty Acid Composition of the Algae, Experimental Diets, and Fish

The lipid fractions were dissolved in toluene, and the fatty acids methyl esters (FAMES) were obtained by transesterification with sulfuric acid (1%) in methanol [23]. The reaction occurred at 50 °C for 16 h in a dark and molecular nitrogen atmosphere. Methyl esters were extracted with hexane:diethyl ether (1:1, *v*/*v*), washed with a solution of KHCO_3_ (2% *w*/*v*), and purified in a column of NH_2_ Sep-pack (Waters, Mildford, MA, USA) with hexane as a solvent. The FAMES were separated by Gas Chromatography using helium as a carrier gas in a GC column BPX70 (70% cyanopropylpolysilphenylene-siloxane) 60 m × 0.25 mm × 0.25 mm (SGE analytical science) (Thermo Fisher Scientific, Madrid, Spain). The initial column temperature was 140 °C for 10 min. Then it was raised to 240 °C at a rate of 2.5 °C min^−1^ and held at 240 °C for 10 min. FAMES detection was made through a flame detector, and the peaks were identified with a standard FAMES pattern (Supelco 37 comp. 47885-U).

### 2.8. Evaluation of the Intestinal Microbiota

#### 2.8.1. DNA Extraction, PCR Amplification, and Analysis of DGGE Patterns

Total DNA was extracted from samples taken at day 0, 7, 15, and 30 according to Martínez et al. [24], with some modifications described by Tapia-Paniagua et al. [25]. The quality of the DNA was determined using agarose gel electrophoresis (1.5%) in the presence of ethidium bromide. DNA was amplified using the 16S rDNA bacterial domain-specific primers 968-GC-F (5′GAACGCGAAGAACCTTAC-3′) and 1401-R (5′CGGTGTGTACAAGACCC-3′) [26], targeting V6-V8 regions of 16S rDNA. PCR was performed as previously described by Tapia-Paniagua et al. [25]. PCR products were separated by denaturing gradient gel electrophoresis (DGGE) in a Dcode TM system (Bio-Rad Laboratories, United States), according to Rico et al. [27]. Gels were stained with AgNO_3_ after electrophoresis [28]. DGGE patterns of all samples were performed twice, which were analyzed using FPQuest Software version 4.0 (Applied Maths BVBA, Sint-Martens-Latem, Belgium). 

Pearson correlation was used to calculate similarity indices from densitometric curves of the scanned DGGE profiles [29]. The generation of dendrograms employing the Unweighted Pair Groups Method with Arithmetic Averages (UPGMA) was used to cluster DGGE patterns. The microbial communities of the intestine were studied according to Rico et al. [27] and Abdala-Díaz et al. [30], assessing (i) the species richness (R), (ii) the range-weighted richness (Rr), (iii) the Pareto-Lorenz distribution curves, and (iv) the Gini coefficient.

#### 2.8.2. Sequencing of DGGE Bands

The predominant bands in the DGGE gels were extracted and reamplified as previously described. The High Pure Spin Kit PCR purification kit was used to purify the products (Roche, Basel, Switzerland). The amplicons were sequenced on an ABI PRISM 377 sequencer (PerkinElmer, Waltham, MA, USA), and the nucleotide sequences were submitted to a BLAST search in GenBank (http://blast.ncbi.nlm.nih.gov/Blast.cgi, accessed on 7 September 2022) and a SINA alignment in the SILVA database (https://www.arb-silva.de/aligner/, accessed on 8 September 2022) to retrieve the closest known alignment identities for the incomplete 16S rRNA sequences [31,32]. Species-level identification was accepted at ≥97% nucleotide alignment. The Ribosomal Database Project’s CHECK CHIMERA tool was used to identify chimeric sequences [33]. The ARB software tool, Version 6.0.3 [34] was used to perform phylogenetic studies on clone libraries, and sequences were matched using the positional tree server with a data set comprising the closest related matches from the Ribosomal Database Program.

### 2.9. Statistical Analysis

Obtained values are expressed as means ± standard deviations (SD). Normal distribution was checked for all data with the Shapiro–Wilk test, while the homogeneity of the variances was obtained using the Levene test. When necessary, an arcsine transformation was performed. One-way analysis of variance (ANOVA) followed by Tukey’s Test was employed to determine the statistical differences between different experimental diets using the software STATISTICA version 7.0. Statistical significance of mean differences was considered to be attained with *p* < 0.05.

## 3. Results

### 3.1. Growth Performance, Nutrient Utilization, and Proximate Composition

No fish mortality occurred during the experimental period. The overall growth performance and nutrient utilization data are shown in Table 3. Dietary treatments that included the microalgal blend (MB-5, MB-15, and MB-25) tended to reduce WGR, SGR, and FCR compared with the control group, although differences were not significant (*p* > 0.05). Conversely, PER and CY were higher in specimens fed with diets including microalgae meal than in the control group, although no significant differences were observed (*p* > 0.05). Regarding HSI, VI, and K, no significant differences were observed between dietary treatments (*p* > 0.05). 

The proximate composition of *S. aurata* specimens is presented in Table 4. The body composition of fish did not show any significant variation between treatments, except for fish fed on MB-15 and MB-25 that exhibited significantly lower lipid content than other experimental groups (*p* < 0.05).

### 3.2. Fatty Acid Composition of Fish

The liver fatty acid composition of *S. aurata* juveniles fed experimental diets for 30 days is presented in Table 5. The results revealed that microalgae inclusion significantly affected the fatty acid profile, especially with the highest dietary microalgal inclusion level. The saturated fraction (SFA) was significantly lower in fish fed on MB-15 and MB-25 diets (*p* < 0.05), mainly due to the lower value for stearic acid (18:0) observed in this fraction. On the contrary, total polyunsaturated fatty acids (PUFA) tended to increase in fish fed on microalgae-supplemented diets, although significant differences were only observed in fish fed on the MB-25 diet. Docosahexaenoic acid (DHA, 22:6n3) content was significantly higher in fish fed on MB-15 and MB-25 diets, reaching values above 15% of the total FAMEs (15.6% and 15.8%, respectively; *p* < 0.05). The proportion of eicosapentaenoic acid (EPA, 20:5n3) was significantly higher in MB-25-fed fish compared to the control group (*p* < 0.05). MB-15 and MB-25 fish showed significant n-3 fatty acids content and higher n3/n6 ratio (*p* < 0.05).

The muscle fatty acid composition of *S. aurata* juveniles is presented in Table 6. Polyunsaturated fatty acids (PUFA) were the predominant lipids in muscle tissue (42.3–49.6%), followed by saturated (SFA) (27.9–33.3%) and monounsaturated fatty acids (MUFA) (24.4–25.2%), regardless of dietary treatment. Similar to that observed in hepatic tissue, the main effect of microalgae inclusion was the significant increase in the muscle PUFA content and the decrease in SFA fraction (*p* < 0.05), whereas MUFA content remained unaffected (*p* > 0.05). The inclusion of microalgae induced a significant reduction of myristic acid (C14:0) and palmitic acid (C16:0) compared to control fish (*p* < 0.05). On the other hand, the proportion of ARA and DHA was significantly higher in fish fed the higher microalgae inclusion levels (MB-15 and MB-25 diets), reaching values above 11% and 27% of the total fatty acids, respectively (*p* < 0.05). 

### 3.3. Evaluation of Intestinal Microbiota

**Figure 1:**
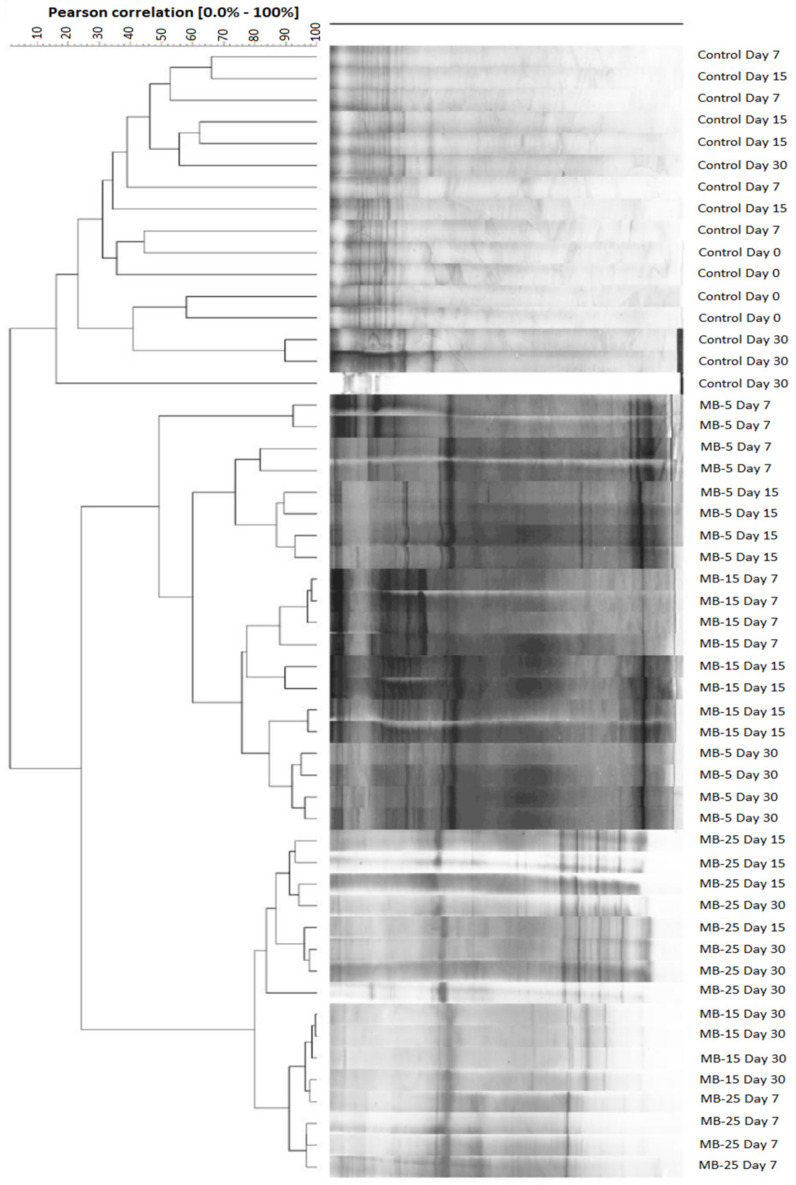
Clustering based on pairwise similarity index of DGGE patterns obtained from intestinal samples of juvenile *S. aurata* fed experimental diets for 30 days. Codes are: MB-5: 5% microalgae blend meal inclusion; MB-15: 15% microalgae blend meal inclusion; MB-25: 25% microalgae blend meal inclusion.

A clustering analysis of the PCR-DGGE patterns from intestinal microbiota revealed two different clusters. The first corresponded to the Control group at different sampling times. The second corresponded to the specimens fed with diets including microalgae at all sampling times, showing a similarity index of less than 10% (Figure 1). Within the Control group, no defined group was observed; on the contrary, the PCR-DGGE patterns appeared mixed regardless of the sampling time. On the other hand, within the group of diets containing microalgal meal, two well-differentiated groups were observed, corresponding to fish fed (1) MB-25 at all sampling times and MB-15 at day 30 (about 80% similarity) and (2) MB-5 at all sampling times and MB-15 at days 7 and 15 (48% similarity). 

Average numbers of species richness (R) as determined by PCR-DGGE bands ranged from 7.75 ± 2.22 to 36.50 ± 6.40 bands in all intestinal samples (Table 7). Significant differences were observed in species richness at all sampling times, where fish fed MB-25 were statistically higher than the other groups (Table 7; *p* < 0.05). On the other hand, species richness of the MB-15 group varied over time, observing a significant reduction at day 15 (7.75 ± 2.22; *p* < 0.05). The distribution (evenness) of bacterial species within the control and the MB groups were not significantly different on the sampling days, except for day 30, on which the MB-15 diet was significantly reduced (Gini coefficient = 0.11) when compared to the other groups (*p* < 0.05). In addition, range-weighted richness (Rr) was used to study the environment’s carrying capacity based on the number of PCR-DGGE bands and the percentage of denaturing gradient in the DGGE gel needed to describe the total diversity of the sample analyzed. Rr values ranged from 7.97 ± 2.65 to 374.00 ± 180.47 (Table 7). The group fed the MB-25 diet always presented Rr values significantly higher than the rest of the treatments for all times (*p* < 0.05). 

**Figure 2:**
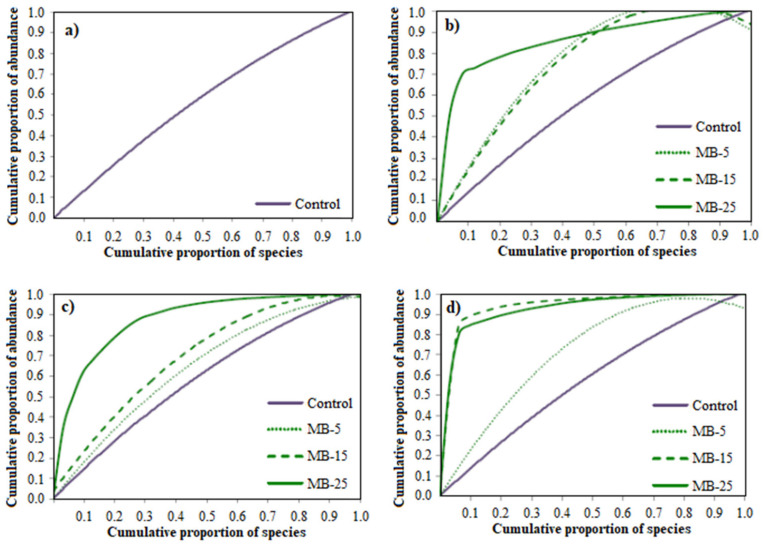
Pareto–Lorenz distribution curves based on PCR-DGGE patterns from intestinal samples) of juvenile *S. aurata* fed experimental diets for 30 days at day 0, 7, 15, and 30 ((**a**–**d**), respectively). Codes are: MB-5: 5% microalgae blend meal inclusion; MB-15: 15% microalgae blend meal inclusion; MB-25: 25% microalgae blend meal inclusion.

A Pareto–Lorenz distribution curve was constructed to assess the functional organization and evenness of the distribution of the bacterial population in each group investigated. It was observed that 20% (0.2 proportions on the x-axis) of the bacterial species were responsible for 20 to 25% of the functional interaction within the bacterial community structure of the fish fed the control diet (Figure 2). As for diets containing the microalgae blend, MB-5 and MB-15 groups presented a similar distribution curve at days 7 and 15, corresponding to 50% of the accumulated intensities. After 15 days of MB-25 diet administration, 20% of the bacterial species accumulated over 80% of the total intensity of the bands. At day 30, the distribution curves of MB-15 and MB-25 groups corresponded to 90% of accumulated intensities, higher than MB-5 and control groups (40 and 25%, respectively). 

**Figure 3:**
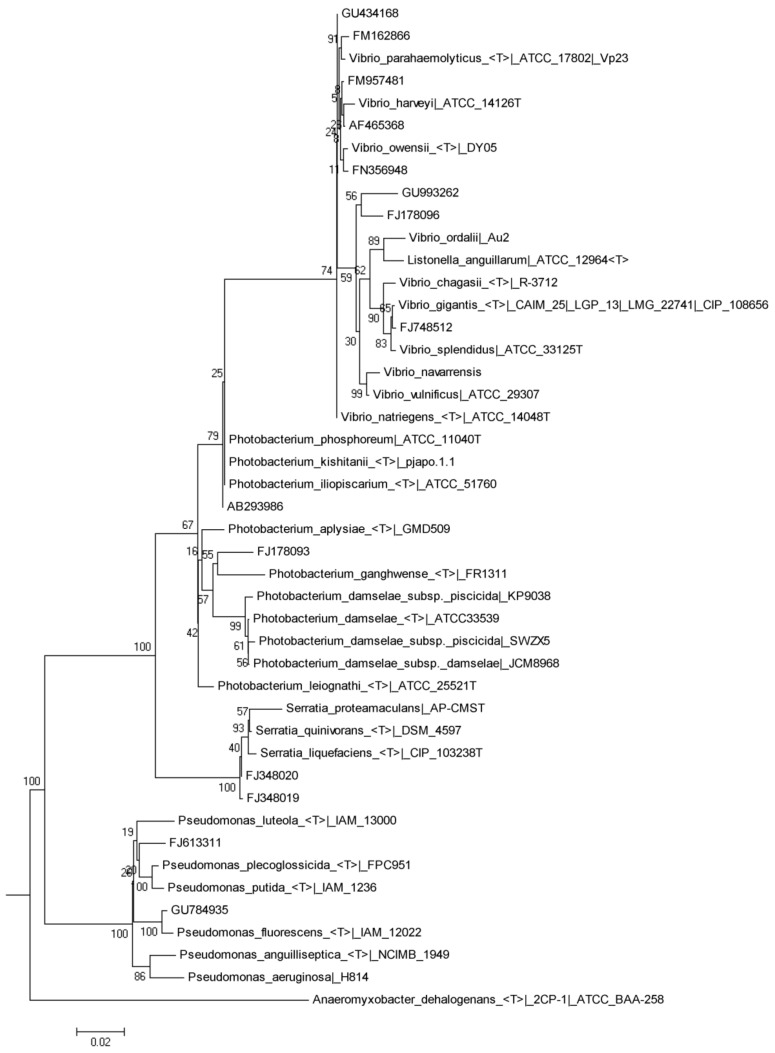
Phylogenetic tree of clusters based on 16S rRNA sequences showing the relationships of the predominant bands obtained from intestinal samples of juvenile *S. aurata* with their closest species of *Vibrio*, *Photobacterium*, *Pseudomonas*, and *Serratia* genera.

The dietary inclusion of the microalgae blend induced changes in the fish’s predominant bacteria of the intestinal microbiota (Table 8). The predominant bands sequenced from the PCR-DGGE patterns of the fish fed with the control diet for the different times corresponded to an uncultured cyanobacterium and to isolates of the genera *Vibrio* and *Photobacterium*, phylogenetically related to *Vibrio alginolyticus*, *Vibrio harveyi*, and *Vibrio* sp., and *Photobacterium* sp., respectively. A bacterium from the *Enterobacteriaceae* group phylogenetically related to *Serratia proteomaculan* was also sequenced (Figure 3). The inclusion of microalgae blend meal produced the appearance of new predominant bands. Thus, sequences related to the genera *Shewanella* and *Comamonas* were obtained, as well as lactic acid bacteria (LAB), such as *Lactobacillus delbrueckii*. A band related to the *Thalassomonas* genus also appeared as a predominant group of the intestinal microbiota of fish fed the MB-25 diet at days 15 and 30. At day 30, it was also observed that MB-15 and MB-25 diets increased the number of bands related to the genus *Vibrio*, phylogenetically related to species such as *V. harveyi*, *Vibrio ichthyoenteri*, *Vibrio ordalii*, *Vibrio parahaemolyticus* and *Vibrio vulnificus* (Figure 3).

## 4. Discussion

### 4.1. Growth Performance, Nutrient Utilization, and Proximate Composition

Fishmeal usage in diets for carnivorous fish has drawn criticism for the aquaculture sector. However, using alternative protein sources has the potential to reduce important nutrients such as essential amino acids and highly unsaturated fatty acids, which might have detrimental impacts on general fish health [35], and lead to a product with worse nutritional quality for the consumer. Furthermore, consumers expect high-quality products, which is frequently confronted by the economic requirement of producing more economical aquafeeds to operate a more lucrative business [36], which may be accomplished by employing low-cost alternative feedstuffs. Our results prove that replacing up to 25% of fishmeal with microalgae blend in feeds for *S. aurata* is possible without affecting survival or growth. 

Due to their well-balanced nutritional composition, microalgae meals are becoming more and more significant in the aquafeed business [8]. For instance, the combination of *Schizochytrium limacinum* meal and *Haematococcus pluvialis* lipid-extracted meal could replace fishmeal by 80% without producing any negative effects on long-fin yellowtail (*Seriola rivoliana*) [37]. According to Vizcaíno et al. [15], including *S. almeriensis* meal (up to 39%) in practical diets had no discernible negative impacts on gilthead seabream’s growth performance (*S. aurata*). For several species, however, only a small percentage of microalgal replacement led to growth outcomes comparable to those of control diets. For European sea bass (*Dicentrarchus labrax*), a 20% fishmeal replacement of *Isochrysis* sp. had no negative effect on feed intake or growth performance [38]. For Atlantic salmon (*Salmo salar*), only 6% of fishmeal replacement by *Phaeodactylum tricornutum* was recommended to avoid adverse effects on feed conversion ratio (FCR) and digestibility [39]. Since higher levels of fishmeal replacement reduced growth performance, only 5% of the freeze-dried biomass of *Tetraselmis suecica* was indicated for *S. aurata* [13]. Thus, it seems that the farmed fish species and the microalgae meal have an impact on fish development in response to fishmeal replacement with microalgae meals. 

In the present study, although not significantly, the hepatosomatic index (HSI) decreased in microalgae-fed specimens, especially with high inclusion levels. Similar results were obtained by other authors [13,15] who detected that high inclusion of microalgae meal induced lower HSI compared to microalgae-free control diets. According to Nakagawa [40] and Ergün et al. [41], this finding appears to indicate that the better effectiveness of mobilizing lipids in the fish liver is thought to be directly correlated with the quantity of microalgae inclusion. As a result, the higher lipid mobilization efficiency shown in fish given the highest levels of microalgae appears to be associated with the lower HSI values, which may change the fish’s body lipid content [42].

Proximate body composition means the determination of the fish’s water, protein, fat, and ash content [43], which is thought to be a reliable predictor of its health and physiological state [44]. Although none of the diets modified protein content, fish fed 15% and 25% microalgae blends showed statistically decreased body lipids. By enhancing the utilization of lipids as an energy source in fish metabolism, increased lipid mobilization can decrease its storage [13,15]. This mechanism might explain our findings in *S. aurata* specimens fed on microalgae diets.

### 4.2. Fatty Acid Composition of Fish

Since fishmeal was replaced with microalgae, it is essential to ensure that the animal’s essential fatty acid requirements were met. The evaluation of fatty acid composition offers relevant information about the feed’s influence on the fish’s chemical profile [45]. According to Fountoulaki et al. [46], dietary fatty acid composition changes are reflected in the fatty acid composition of marine fish tissues. As expected, the significant reduction of the proportion of SFA and the increase in the incidence of 18:1n9 observed in the liver and muscle tissue of fish fed on microalgae-supplemented diets reflected the fatty acid profile of experimental feeds. Tibaldi et al. [38] pointed out that dietary use of microalgae seemed responsible for a certain degree of selective retention of specific fatty acids in fish tissues. In this regard, the results obtained in the present study revealed that the proportion of EPA and DHA in the liver and ARA and DHA in the muscle of fish fed the microalgae-supplemented diets were significantly higher than their respective dietary concentrations. Inadequate intake of these fatty acids, which are essential for cellular membrane structure and function, results in decreased growth and increased fish mortality as well as other pathologies such as liver or intestine steatosis [47].

The observed selective retention has been previously reported in other marine fish species fed with algae-supplemented diets [10,48,49], pointing to a relationship between the algae inclusion level and the higher efficiency of mobilization of lipids in fish liver. Indeed, fish fed the higher level of microalgae tended to show low HSI values, though no significant difference was found. It is widely known that EPA and DHA have different physiological functions and can be metabolized and incorporated into tissues differently [50]. Bell et al. [51] and Tocher [52] related this phenomenon to the higher catabolic use of EPA and/or to preferential retention of ARA and DHA during the biosynthesis and remodeling of phospholipids. Other authors, such as Peng et al. [53] and Alhazzaa et al. [54], pointed out that dietary fatty acid composition could affect the gene expression and activities of specific enzymes involved in lipid metabolism yielding the accumulation of specific fatty acids in the fish tissues. Therefore, these results indicate noticeable effects of dietary inclusion of a microalgae blend on fish lipid metabolism, though the processes concerning this relationship remain to be fully ascertained.

### 4.3. Evaluation of Predominant Intestinal Microbiota

Given the functional role of the gut microbiome in host physiology, general development, and health, microbiome regulation may be a practical way to reduce fish diseases in aquaculture [55]. Dietary changes in the microbiome may also impact the immunological response, as well as fish susceptibility and possible resistance to diseases [56]. Here, we reported that the effects of the microalgae blend meal on the gut microbiome of gilthead seabream significantly varied depending on the inclusion levels.

The dendrogram from the cluster analysis of the intestinal microbiota showed two well-differentiated clusters (control and specimens fed with microalgae) after 7 days of feeding, indicating that the microalgae blend supplementation modified the intestinal microbiota. These results are consistent with those published by Abdala-Díaz et al. [30], who reported comparable differentiated clusters after 7 days of feeding the control and 25% *Ulva rigida* diet. The specific richness (R) and Rr values were systematically higher in fish-fed 25% microalgae blend at all sampling times. Moreover, at the end of the feeding trial, Rr values were higher than 30 in all groups fed microalgae meal, reflecting a habitable environment in which many different microorganisms can appear [57], thus allowing quick adaptability to new conditions such as stress processes, temperature, and salinity changes, or pathogen infection [58]. Studies considering the influence of dietary microalgae on fish intestinal microbial communities reported confounding results. According to Zhang et al. [59], dietary *Chlorella* meal did not affect microbial diversity and richness species of largemouth bass (*Micropterus salmoides*) when the level of fishmeal replacement was less than 50%. In contrast, higher replacement levels resulted in a remarkable diversity loss, indicating different gut microbes’ responses to dietary inclusion levels. A reduction in intestinal microbial species richness and diversity was found in *S. aurata* fed a commercial feed supplemented with 10% *Tetraselmis chuii* or *P. tricornutum* [60]. 

On the other hand, some research has found that low-level dietary microalgae supplementation (ranging from 5 to 15%) results in an increasing trend in microbial diversity [61,62]. Our results indicate that the microalgae blend promoted an increase in bacterial species diversity and a distinct shift in microbiota fingerprinting as inclusion levels increased, implying an adaptive response to the dietary formulations and consistent with Lyons et al. [61]. Thus, a more diversified microbiota in the microalgae-fed fish might reflect additional plasticity in the microbiome to help break down, digest, and absorb the microalgal meal contained in their diet.

At the taxonomical level, the results showed that *Photobacterium* species were found among the detected DGGE bands of all experimental groups but not in any microalgae-fed groups at the end of the trial. This genus includes pathogenic fish species [63], whereas others have been isolated from the intestinal microbiota of *S. aurata* [27]. In the case of fish fed microalgae, predominant DGGE bands associated with the genus *Photobacterium* were not detected at day 30, which is consistent with the results of Abdala-Díaz et al. [30]. These authors did not identify DGGE bands related to *Photobacterium* species after 30 days of feeding with 25% of *U. rigida*. On the contrary, Tapia-Paniagua et al. [64] reported the presence of *Photobacterium* species in the microbiota of Senegalese sole (*Solea senegalensis*) fed 5% *Ulva ohnoi*. These results may indicate that the prevalence of this genus in the microbiota seems to be influenced by algal species.

Although the presence of specifical *Vibrio* species changed with the diet, *Vibrio* pathogenic species (mainly *V. alginolyticus*, *V. harveyi*, *V. ichthyoenteri*, and *V. parahaemolyticus*) appeared in the intestinal microbiota of all groups. However, in our study, *Vibrio* seems not to play a pathogenic role according to the absence of fish mortality and disease symptoms.

DGGE bands associated with *Lactobacillus delbrueckii* subspecies, specifically *L. delbrueckii* subsp *bulgaricus*, were discovered to be prevalent in the DGGE patterns of fish given MB-15 and MB-25 diets at day 30. Other studies have reported the same species in seabream fed diets enriched with *U. rigida* after 30 and 60 days of feeding [27,30]. Mohammadian et al. [65] proposed that increased colonization of *L. delbrueckii* might be due to higher probiont adhesion ability to the intestinal mucosa [66] or lower intestinal pH generated by *Lactobacillus* species [67], which suppresses pathogenic bacterial growth [68]. Furthermore, *Lactobacillus* species, including *L. delbrueckii*, have been shown to stimulate the innate immune response, which enhances fish resistance to diseases [65,69]. Interestingly, *Lactobacillus* species have antimicrobial activity against fish, and food-borne pathogens [70], including species of the *Enterobacteriaceae* family, which could be the cause of the absence of DGGE bands related to that family in fish fed MB-25 diets.

Some strains of the genus *Pseudoalteromonas* were promoted after 30 days in fish fed the MB-25 diet. In this sense, this genus includes strains with inhibitory capacity against fish-pathogenic *Vibrio* and *Photobacterium* strains [71], as well as against Gram-positive marine bacteria [72]. Thus, the promotion of *Pseudoalteromonas* could be related to the reduction in the presence of DGGE bands corresponding to *V. harveyi* and *Photobacterium* genus. Furthermore, they may also be used as a defense agent by the microbiota and marine fauna, suggesting that it is probiotic, at least in certain species [73]. 

*Shewanella* is an opportunistic pathogen affecting various fish species [74,75]. Several studies, however, have found that *Shewanella* species are helpful to the host’s intestinal health [76]. Furthermore, it has been characterized as a probiotic for farmed fish species such as *S. senegalensis* and *S. aurata* [76]. In our study, *Shewanella* was found in all microalgae diet groups, not the control group. On the contrary, Ma et al. [77] reported a reduction in the abundance of *Shewanella* in zebrafish (*Danio rerio*) fed with any of *Schizochytrium* sp., *A. platensis*, *Chlorella sorokiniana*, *Chromochloris zofingiensis*, and *Dunaliella salina*. It might be suggestive to think that the promotion/reduction in the abundance of *Shewanella* species may increase or decrease depending on the varied inclusion levels, the specific microalgae strain and fish species, and/or the duration of the experiment.

These results lead us to believe that the dietary inclusion of a microalgae blend for 30 days, especially a 25% inclusion, promotes an optimal microbial diversity state.

## 5. Conclusions

Overall, *S. aurata* juveniles adapted well to the nutritional formulation used in this study since survival and growth were comparable to the control group after 30 days of feeding. According to the results, a 25% microalgae blend promoted appropriate microbial diversity, favoring the presence of potentially beneficial bacteria, such as *Lactobacillus*, and significantly influencing fish lipid metabolism. However, more study is required to determine if the effects appear during long-term studies for attaining a compromise with fish development and assessing the response against fish pathogens.

## Figures and Tables

**Table 1:** Ingredient composition and nutrient composition of experimental diets.

Ingredients (g kg^−1^ DM)	Control	MB-5	MB-15	MB-25
Fishmeal (LT-94)	650	615	543	471
*Microalgae blend* ^a^	0	50	150	250
Fish oil	32	30	26	22
Soybean lecithin ^b^	10	10	10	10
Maltodextrin ^b^	245	232	208	184
Cellulose ^c^	20	20	20	20
Choline chloride ^c^	8	8	8	8
Vitamins and minerals ^d^	25	25	25	25
Sodium alginate ^d^	10	10	10	10
Proximate composition (% dry matter)				
Crude protein	45.2	44.8	44.9	45.7
Crude lipid	12.4	12.0	12.1	11.9
Ash	10.8	10.9	11.1	11.2
Moisture	6.7	7.2	7.1	7.5

Codes are: MB-5: 5% microalgae blend meal inclusion; MB-15: 15% microalgae blend meal inclusion; MB-25: 25% microalgae blend meal inclusion. ^a^ Microalgae blend: 49.5% crude protein, 21.3% total carbohydrates, 12.6% crude fat, and 13.3% ash (% dry matter). ^b^ Emilio Peña S.A., aditivos alimentarios, Torrent, Valencia. Spain. ^c^ Sigma-Aldrich, Madrid. Spain. ^d^ Vitamin and mineral premix (values are g kg^−1^ except to those in parenthesis): Premix: 25; Choline. 10; DL-a-tocoferol. 5; ascorbic acid. 5; (PO_4_)_2_Ca_3_. 5. Premix composition: retinol acetate. 1,000,000 IU kg^−1^; calciferol. 500 IU kg^−1^; DL-a-tocoferol. 10; menadione sodium bisulfite. 0.8; thiamin hydrochloride. 2.3; riboflavin. 2.3; pyridoxine hydrochloride. 15; cyanocobalamin. 25; nicotinamide. 15; pantothenic acid. 6; folic acid. 0.65; biotin. 0.07; ascorbic acid. 75; inositol. 15; betaine. 100; polypeptides. 12; Zn. 5; Se. 0.02; I. 0.5; Fe. 0.2; CuO. 15; Mg. 5.75; Co. 0.02; Met. 1.2; Cys. 0.8; Lys. 1.3; Arg. 0.6; Phe. 0.4; Tryp. 0.7.

**Table 2:** Fatty acid composition (% fatty acids) of microalgae blend and experimental diets used in the feeding trial.

Fatty Acids	Experimental Diets				*Microalgae* *blend*
	Control	MB-5	MB-15	MB-25	
Myristic acid (C14:0)	7.20	3.81	3.87	3.98	2.81
Palmitic acid (C16:0)	35.63	30.21	28.92	24.45	13.22
Palmitoleic acid (C16:1)	3.94	7.31	7.41	7.32	6.32
Stearic acid (C18:0)	9.07	5.33	4.28	3.89	3.78
Oleic acid (C18:1n9)	6.52	15.79	16.23	16.42	15.85
Linoleic acid (C18:2n6)	5.93	7.39	6.92	7.88	10.70
α-linolenic acid (C18:3n3)	0.81	1.72	1.91	2.14	5.18
Eicosenoic acid (C20:1)	0.83	1.27	0.89	1.12	1.12
Eicosatrienoic acid (C20:3n3)	0.40	0.37	0.41	0.49	1.31
Arachidonic acid (ARA; C20:4n6)	0.25	0.38	0.41	0.39	2.44
Eicosapentaenoic acid (EPA; C20:5n3)	8.17	9.10	9.26	10.48	12.88
Eurcic acid (C22:1n9)	0.64	1.02	1.11	2.07	4.17
Docosahexaenoic acid (DHA; C22:6n3)	12.10	12.53	13.20	15.09	16.33
Nervonic acid (C24:1)	0.72	0.42	0.38	0.31	0.92
Saturated fatty acid (SFA)	59.69	42.70	41.90	36.29	22.78
Monounsaturated fatty acid (MUFA)	12.65	25.81	26.02	27.24	28.38
Polyunsaturated fatty acid (PUFA)	20.66	24.49	25.11	29.47	48.84
n3	21.48	23.72	24.78	28.20	35.70
n6	6.18	7.77	7.33	8.27	13.14
n9	7.16	16.81	17.34	18.49	20.02
n3/n6	3.47	3.05	3.38	3.40	2.71
EPA/DHA	0.67	0.72	0.69	0.69	0.78

Codes are: MB-5: 5% microalgae blend meal inclusion; MB-15: 15% microalgae blend meal inclusion; MB-25: 25% microalgae blend meal inclusion.

**Table 3:** Growth performance, nutrient utilization, and somatic indices of juvenile *S. aurata* fed experimental diets for 30 days.

Parameters	Control	MB-5	MB-15	MB-25	*p*
Growth and nutrient utilization					
Initial mean weight (g)	15.9 ± 0.2	16.6 ± 0.6	15.9 ± 0.7	16.0 ± 1.3	n.s.
Final mean weight (g)	28.7 ± 1.3	28.5 ± 2.3	27.2 ± 0.7	26.9 ± 1.6	n.s.
WGR (%)	75.8 ± 9.1	71.7 ± 8.8	71.7 ± 6.5	68.8 ± 4.2	n.s.
SGR (% day^−1^)	2.0 ± 0.2	1.9 ± 0.2	1.9 ± 0.1	1.8 ± 0.1	n.s.
Total feed intake (g fish^−1^)	17.9 ± 1.8	15.5 ± 2.9	14.7 ± 0.9	15.3 ± 2.2	n.s.
FCR	1.4 ± 0.2	1.3 ± 0.3	1.3 ± 0.2	1.4 ± 0.2	n.s.
PER	1.1 ± 0.1	1.1 ± 0.1	1.1 ± 0.2	1.2 ± 0.1	n.s.
CY (%)	86.3 ± 3.8	90.9 ± 8.1	87.1 ± 1.6	89.2 ± 2.5	n.s.
Somatic indices					
HSI (%)	1.9 ± 0.1	1.8 ± 0.3	2.0 ± 0.4	1.6 ± 0.3	n.s.
VI (%)	13.7 ± 3.8	9.1 ± 8.1	12.9 ± 1.6	10.8 ± 2.5	n.s.
K (%)	1.8 ± 0.1	1.9 ± 0.2	1.8 ± 0.1	1.9 ± 0.1	n.s.

Values are expressed as mean ± SD (n = 3). Codes are: MB-5: 5% microalgae blend meal inclusion; MB-15: 15% microalgae blend meal inclusion; MB-25: 25% microalgae blend meal inclusion. n.s.: not significant.

**Table 4:** Carcass composition (% in dry weight basis) of juvenile *S. aurata* specimens fed experimental diets for 30 days.

Parameters	Control	MB-5	MB-15	MB-25	*p*
Protein	60.4 ± 0.7	59.3 ± 0.4	59.9 ± 0.6	59.0 ± 1.6	n.s.
Lipid	19.0 ± 0.4 ^a^	19.8 ± 1.6 ^a^	12.5 ± 1.4 ^c^	15.4 ± 1.7 ^b, c^	0.023
Ash	15.3 ± 0.2	15.4 ± 0.7	15.6 ± 0.4	15.1 ± 0.7	n.s.

Values are expressed as mean ± SD (n = 6). Values in the same row with different lowercase letters indicate significant differences (*p* < 0.05). Codes are: MB-5: 5% microalgae blend meal inclusion; MB-15: 15% microalgae blend meal inclusion; MB-25: 25% microalgae blend meal inclusion. n.s.: not significant.

**Table 5:** Liver fatty acid composition (% fatty acids) of juvenile *S. aurata* fed experimental diets for 30 days.

Fatty Acids	Control	MB-5	MB-15	MB-25	*p*
Myristic acid (C14:0)	3.65 ± 0.11	3.58 ± 0.14	3.39 ± 0.21	3.08 ± 0.24	n.s.
Palmitic acid (C16:0)	19.04 ± 0.24	19.11 ± 0.29	19.23 ± 0.92	20.94 ± 0.78	n.s.
Stearic acid (C18:0)	16.63 ± 0.23 ^a^	16.03 ± 0.29 ^a^	12.83 ± 0.35 ^b^	10.81 ± 0.69 ^c^	0.016
Oleic acid (C18:1n9)	19.87 ± 0.46 ^b^	18.91 ± 0.31 ^c^	22.03 ± 1.23 ^a^	22.54 ± 1.41 ^a^	0.032
Linoleic acid (C18:2n6)	5.88 ± 0.21	5.74 ± 0.19	5.79 ± 0.96	5.73 ± 0.43	n.s.
α-linolenic acid (C18:3n3)	1.20 ± 0.03 ^b^	1.83 ± 0.09 ^a^	0.85 ± 0.12 ^c^	0.95 ± 0.10 ^c^	0.011
Eicosanoic acid (C20:0)	0.91 ± 0.08 ^a^	0.95 ± 0.05 ^a^	0.41 ± 0.09 ^b^	0.39 ± 0.06 ^b^	<0.001
Eicosatrienoic acid (C20:3n6)	2.36 ± 0.17 ^a^	2.21 ± 0.16 ^a^	1.68 ± 0.11 ^b^	1.59 ± 0.06 ^b^	0.024
Arachidonic acid (ARA; C20:4n6)	7.45 ± 0.11	7.67 ± 0.09	8.14 ± 1.25	8.15 ± 0.99	n.s.
Eicosapentaenoic acid (EPA; C20:5n3)	6.43 ± 0.28 ^b^	6.35 ± 0.25 ^b^	6.53 ± 0.11 ^b^	7.04 ± 0.16 ^a^	0.036
Behenic acid (C22:0)	2.10 ± 0.23 ^a^	1.95 ± 0.18 ^b^	1.31 ± 0.02 ^c^	0.39 ± 0.03 ^d^	<0.001
Erucic acid (C22:1n9)	0.13 ± 0.06	0.16 ± 0.08	0.25 ± 0.09	0.21 ± 0.03	n.s.
Docosahexaenoic acid (DHA; C22:6n3)	12.11 ± 0.42 ^b^	12.98 ± 0.33 ^b^	15.62 ± 0.41 ^a^	15.75 ± 0.34 ^a^	0.039
Lignoceric acid (C24:0)	2.05 ± 0.11	2.30 ± 0.08	2.11 ± 0.13	2.14 ± 0.21	n.s.
Nervonic acid (C24:1)	0.19 ± 0.07 ^b^	0.23 ± 0.06 ^a^	0.25 ± 0.05 ^a^	0.29 ± 0.04 ^a^	0.041
Saturated fatty acid (SFA)	44.38 ± 0.34 ^a^	43.92 ± 0.38 ^a^	39.28 ± 1.03 ^b^	37.75 ± 0.93 ^b^	0.018
Monounsaturated fatty acid (MUFA)	20.19 ± 0.55 ^b^	19.30 ± 0.55 ^b^	22.53 ± 1.47 ^a^	23.04 ± 1.11 ^a^	0.009
Polyunsaturated fatty acid (PUFA)	35.43 ± 0.65 ^c^	36.78 ± 0.65 ^c^	38.19 ± 0.35 ^b^	39.21 ± 0.28 ^a^	0.010
n3	19.74 ± 0.75 ^b^	21.16 ± 0.75 ^a, b^	22.58 ± 1.18 ^a^	23.74 ± 0.86 ^a^	0.016
n6	15.69 ± 0.43	15.62 ± 0.43	15.61 ± 1.23	15.47 ± 1.13	n.s.
n9	20.00 ± 0.50 ^b^	19.07 ± 0.35 ^c^	22.28 ± 1.30 ^a^	22.75 ± 1.41 ^a^	0.034
n3/n6	1.25 ± 0.17 ^c^	1.35 ± 0.17 ^b, c^	1.44 ± 0.03 ^b^	1.53 ± 0.03 ^a^	0.041
EPA/DHA	0.53 ± 0.03 ^a^	0.48 ± 0.01 ^b^	0.41 ± 0.06 ^c^	0.44 ± 0.02 ^c^	0.044

Values are expressed as mean ± SD (n = 6). Values in the same row with different lowercase letters indicate significant differences (*p* < 0.05). Codes are: MB-5: 5% microalgae blend meal inclusion; MB-15: 15% microalgae blend meal inclusion; MB-25: 25% microalgae blend meal inclusion. n.s.: not significant.

**Table 6:** Muscle fatty acid composition (% fatty acids) of juvenile *S. aurata* fed experimental diets for 30 days.

Fatty Acids	Control	MB-5	MB-15	MB-25	*p*
Myristic acid (C14:0)	4.50 ± 0.06 ^a^	4.11 ± 0.17 ^b^	1.98 ± 0.17 ^c^	1.90 ± 0.14 ^c^	<0.001
Palmitic acid (C16:0)	16.83 ± 0.40 ^a^	16.62 ± 0.26 ^a^	16.41 ± 0.39 ^a^	15.39 ± 0.31 ^b^	0.035
Stearic acid (C18:0)	4.43 ± 0.19 ^c^	4.63 ± 0.25 ^c^	6.42 ± 0.10 ^a^	5.56 ± 0.16 ^b^	0.008
Oleic acid (C18:1n9)	19.83 ± 0.14 ^c^	19.69 ± 0.17 ^c^	22.58 ± 0.19 ^b^	23.45 ± 0.18 ^a^	0.034
Linoleic acid (C18:2n6)	4.92 ± 0.09 ^b^	5.30 ± 0.09 ^a^	3.09 ± 0.22 ^c^	3.00 ± 0.22 ^c^	0.016
α-linolenic acid (C18:3n3)	1.36 ± 0.09 ^a^	1.37 ± 0.05 ^a^	0.99 ± 0.13 ^b^	0.96 ± 0.12 ^b^	0.021
Eicosanoic acid (C20:0)	1.28 ± 0.07 ^b^	1.43 ± 0.08 ^a^	0.48 ± 0.06 ^c^	0.46 ± 0.04 ^c^	0.017
Gondoic acid (C20:1n9)	3.86 ± 0.66 ^a^	4.48 ± 0.72 ^a^	0.82 ± 0.19 ^b^	0.88 ± 0.19 ^b^	<0.001
Eicosatrienoic acid (C20:3n6)	0.48 ± 0.06 ^b^	0.36 ± 0.06 ^c^	0.61 ± 0.04 ^a^	0.57 ± 0.05 ^a, b^	0.031
Arachidonic acid (ARA; C20:4n6)	8.63 ± 0.11 ^b^	8.29 ± 0.12 ^c^	11.95 ± 0.79 ^a^	11.82 ± 0.82 ^a^	0.005
Eicosapentaenoic acid (EPA; C20:5n3)	7.68 ± 0.22	7.29 ± 0.16	7.01 ± 0.51	7.56 ± 0.30	n.s.
Behenic acid (C22:0)	3.98 ± 0.52 ^a^	3.54 ± 0.60 ^a^	2.24 ± 0.63 ^b^	1.60 ± 0.06 ^c^	0.004
Erucic acid (C22:1n9)	0.24 ± 0.09 ^b^	0.28 ± 0.06 ^b^	0.63 ± 0.20 ^a^	0.40 ± 0.08 ^a^	0.031
Docosahexaenoic acid (DHA; C22:6n3)	19.19 ± 0.99 ^b^	19.08 ± 0.89 ^b^	21.57 ± 0.97 ^a^	23.01 ± 1.29 ^a^	0.028
Lignoceric acid (C24:0)	2.23 ± 0.09 ^b^	2.25 ± 0.08 ^b^	2.86 ± 0.12 ^a^	2.96 ± 0.13 ^a^	0.045
Nervonic acid (C24:1)	0.56 ± 0.32 ^a, b^	0.37 ± 0.11 ^b^	0.69 ± 0.16 ^a^	0.48 ± 0.21 ^b^	0.034
Saturated fatty acid (SFA)	33.25 ± 1.01 ^a^	32.58 ± 0.36 ^a^	30.39 ± 0.74 ^b^	27.87 ± 0.53 ^c^	0.022
Monounsaturated fatty acid (MUFA)	24.49 ± 1.11	24.82 ± 0.83	24.39 ± 1.11	25.21 ± 1.05	n.s.
Polyunsaturated fatty acid (PUFA)	42.26 ± 1.10 ^b, c^	41.69 ± 0.97 ^c^	44.51 ± 1.62 ^a, b^	46.92 ± 1.44 ^a^	0.033
n3	28.23 ± 1.39 ^b^	27.74 ± 1.10 ^b^	28.86 ± 1.06 ^b^	31.53 ± 1.08 ^a^	0.039
n6	14.03 ± 0.25 ^b^	13.95 ± 0.08 ^b^	15.65 ± 0.71 ^a^	15.39 ± 0.74 ^a^	0.037
n9	23.93 ± 1.02	24.45 ± 0.94	24.03 ± 0.53	24.73 ± 0.42	n.s.
n3/n6	2.01 ± 0.21	1.98 ± 0.14	1.84 ± 0.16	2.04 ± 0.08	n.s.
EPA/DHA	0.40 ± 0.01	0.38 ± 0.01	0.32 ± 0.09	0.32 ± 0.07	n.s.

Values are expressed as mean ± SD (n = 6). Values in the same row with different lowercase letters indicate significant differences (*p* < 0.05). Codes are: MB-5: 5% microalgae blend meal inclusion; MB-15: 15% microalgae blend meal inclusion; MB-25: 25% microalgae blend meal inclusion. n.s.: not significant.

**Table 7:** Species richness (R), Gini coefficient, and range-weighted richness (Rr) values of intestinal microbiota DGGE patterns of juvenile *S. aurata* fed experimental diets for 30 days.

Day	Diet	R	Gini Coefficient	Rr
0	Control	13.25 ± 1.26 ^b^	0.27 ± 0.01 ^c^	10.44 ± 3.15 ^a, b^
7	Control	13.25 ± 2.63 ^b^	0.23 ± 0.08 ^b^	7.97 ± 2.65 ^a^
	MB-5	12.66 ± 3.79 ^b^	0.21 ± 0.03 ^b^	20.16 ± 2.84 ^c^
	MB-15	11.50 ± 4.04 ^a, b^	0.18 ± 0.03 ^b^	22.28 ± 10.18 ^b, c^
	MB-25	23.33 ± 4.04 ^d^	0.25 ± 0.09 ^b^	136.29 ± 10.74 ^f^
15	Control	15.50 ± 1.29 ^b, c^	0.23 ± 0.05 ^b^	24.72 ± 6.01 ^c^
	MB-5	11.25 ± 3.86 ^a, b^	0.25 ± 0.02 ^b, c^	51.22 ± 4.99 ^d^
	MB-15	7.75 ± 2.22 ^a^	0.18 ± 0.05 ^b^	14.96 ± 5.88 ^b, c^
	MB-25	25.00 ± 5.57 ^d^	0.25 ± 0.05 ^b, c^	292.33 ± 15.03 ^h^
30	Control	13.00 ± 1.00 ^b^	0.23 ± 0.08 ^b^	14.56 ± 3.83 ^b, c^
	MB-5	17.75 ± 2.63 ^c^	0.27 ± 0.03 ^c^	87.79 ± 5.41 ^e^
	MB-15	27.25 ± 3.59 ^d^	0.11 ± 0.04 ^a^	161.67 ± 5.10 ^g^
	MB-25	36.50 ± 6.40 ^e^	0.25 ± 0.05 ^b, c^	374.00 ± 18.47 ^i^
*p*		<0.001	<0.001	<0.001

Values are expressed as mean ± SD (n = 4). Values in the same column with different lowercase letters indicate significant differences (*p* < 0.05). Codes are: MB-5: 5% microalgae blend meal inclusion; MB-15: 15% microalgae blend meal inclusion; MB-25: 25% microalgae blend meal inclusion.

**Table 8:** 16S rDNA sequence similarities to closest relatives of DNA recovered from the respective predominant bands in the DGGE gel corresponding to juvenile *S. aurata* fed experimental diets for 30 days.

				Control				MB-5			MB-15			MB-25		
Closest Relative	Similarity (%)	Genbank Accession Number	Taxon	0 Days	7 Days	15 Days	30 Days	7 Days	15 Days	30 Days	7 Days	15 Days	30 Days	7 Days	15 Days	30 Days
*Agarivorans* sp. QM34	100	GQ426318	γ-proteobacteria	X	X	X	X	X	X	X	X		X	X	X	X
*Comamonas aquatica* strain 530	100	EU841527	β-proteobacteria											X		X
*Enterobacteriaceae bacterium*	99	FJ348019	γ-Proteobacteria			X	X			X			X			
*Lactobacillus delbrueckii* subsp. *bulgaricus*	100	FJ915706	Firmicutes										X		X	X
*Lactococcus* sp.	98	FR873792	Firmicutes										X		X	X
*Photobacterium* sp.	99	FJ178093	γ-proteobacteria			X	X		X		X			X	X	
*Pseudoalteromonas* sp.	98	HM475290	γ-proteobacteria											X		X
*Pseudoalteromonas* sp. DFH4.24	98	FR873779	γ-proteobacteria			X										
*Pseudomonas* sp.	98	GU784935	γ-proteobacteria					X	X	X	X					
*Shewanella* sp.	100	AY515438	γ-proteobacteria					X		X	X	X	X	X	X	X
*Sphingomonas* sp.	98	GU300600	α-Proteobacteria											X		
*Thalassomonas haliotis*	99	AB369381	γ-proteobacteria												X	X
Uncultured *cyanobacterium* clone O7	99	FJ178040	Cyanobacteria	X	X	X	X	X								X
Uncultured *Firmicutes*	100	FM225297	Firmicutes							X		X	X		X	X
*Vibrio alginolyticus*	100	DQ005214	γ-proteobacteria	X		X			X				X		X	X
*Vibrio alginolyticus*	99	EU155510	γ-proteobacteria	X	X	X	X	X		X	X					
*Vibrio alginolyticus* strain UST981101-031	99	EU833999	γ-proteobacteria									X				
*Vibrio communis* strain R-40504	99	GU078675	γ-proteobacteria													X
*Vibrio harveyi* strain BK2	100	HM355956	γ-proteobacteria	X	X			X	X		X		X			
*Vibrio ichthyoenteri*	99	EF635304	γ-proteobacteria								X					
*Vibrio pathogenic* sp.	99	AB274764	γ-proteobacteria													X
*Vibrio ordalii*	97	AF493811	γ-proteobacteria												X	X
*Vibrio parahaemolyticus*	97	EU660313	γ-proteobacteria										X		X	X
*Vibrio* sp.	100	GU434168	γ-proteobacteria	X						X		X	X			X
*Vibrio vulnificus*	100	AY245180	γ-proteobacteria										X			

Codes are: MB-5: 5% microalgae blend meal inclusion; MB-15: 15% microalgae blend meal inclusion; MB-25: 25% microalgae blend meal inclusion.

## Data Availability

Not applicable.
